# Utilization and medical costs of outpatient rehabilitation among children with autism spectrum conditions in Taiwan

**DOI:** 10.1186/s12913-019-4193-z

**Published:** 2019-06-04

**Authors:** Hsing-Jung Li, Chi-Yuan Chen, Ching-Hong Tsai, Chao-Chan Kuo, Kung-Heng Chen, Kuan-Hsu Chen, Ying-Chun Li

**Affiliations:** 1 Department of Child & Adolescent Psychiatry, Kai-Syuan Psychiatric Hospital, No.130, Kaixuan 2nd Road, Lingya District, Kaohsiung City, 802 Taiwan, Republic of China; 2 Department of Rehabilitation, Saint Joseph Hospital, No. 352, Chien Kuo 1st Road, Kaohsiung City, 802 Taiwan, Republic of China; 3 Department of Adult Psychiatry, Kai-Syuan Psychiatric Hospital, No. 130, Kaixuan 2nd Road, Lingya District, Kaohsiung City, 802 Taiwan, Republic of China; 4 Department of Business Management, Institute of Health Care Management, National Sun Yat-Sen University, No. 70, Lienhai Road, Gushan District, Kaohsiung City, 804 Taiwan, Republic of China

**Keywords:** National Health Insurance, Outpatient rehabilitation resources, Autism spectrum condition, Service utilization, Policy

## Abstract

**Background:**

We examined the utilization of rehabilitation resources among children with autism spectrum condition (ASC), a neurodevelopmental condition, in Taiwan.

**Methods:**

We derived from the National Health Insurance Research Database of Taiwan data pertaining to 3- to 12-year-old children for the period 2008–2010. Based on diagnoses executed in accordance with the International Classification of Diseases, Ninth Revision, Clinical Modification, we classified these data into the ASC and non-ASC groups and analyzed them through multiple linear regression model, negative binomial model, independent sample *t* testing, and χ^2^ testing.

**Results:**

Compared with the non-ASC group, the ASC group exhibited higher utilization of rehabilitation resources. Because hospitals are constrained by overall expenditure limits, expenditure on rehabilitation resources has plateaued, preventing any increase in the utilization of rehabilitation resources. In our ASC group, preschool-aged children significantly outnumbered (*p* < 0.001) school-aged children. When stratified by the hospital level, district hospitals reported the highest utilization (*p* < 0.001). When stratified by region, the highest utilization was in Taipei, whereas the lowest was in the East region (*p* < 0.001). The total annual cost, average frequency of visits, utilization of rehabilitation resources, and average cost were all affected by such elements as patient demographics, hospital type and location (*p* < 0.001).

**Conclusions:**

For improving treatment outcomes among children with ASC and decreasing treatment expenditure, policies that promote the timely ASC detection and treatment should be implemented.

## Background

According to a study on disabilities conducted by Taiwan’s Ministry of the Interior in 2010, autism had the fastest growth rate of all disabilities, with an average annual increase of 10.9% since 2009, and the total number of people with autism exceeded 10,000 (Bulletin of the Ministry of the Interior 2011), highlighting that autism spectrum condition (ASC) cannot be ignored. Equivalently, data from clinical rehabilitation services show that the proportion of children with autism has risen annually. ASC is associated with substantial healthcare expenditure and considerable utilization of psychiatric health services [1]. The main treatment were education and psychosocial treatment [2]. Almost children with ASC receive outpatient rehabilitation services instead of hospitalization.

According to the Taiwan’s national health insurance database, the payment of outpatient claims in autism of children and adolescents totaled about 30 millions points in 2000, and it was increased to 130 millions by 2005 [3]. The more complex treatment are, the higher payout point value are based on the NHI payment standard. Because autism in children is a broad-based developmental condition, it can cause varying degrees of developmental delay in terms of gross movements, fine motor movements, self-care, cognitive development, language development, and emotional control. The goal treatment is psychosocial intervention to improve social skills and prosocial behavior with peers which are outpatient rehabilitation services instead of hospital admission treatment. Therefore, the provision of rehabilitation resources for ASC requires considerable manpower from various professional fields. In addition to interventions from physicians (physiatrists, pediatricians, and pediatric psychiatrists) and functional, physical, and speech therapists, clinical psychologists and other therapists are essential to assist patients in achieving greater independence.

As the proportion of children with autism—who also typically have additional medical problems—is rapidly increasing, so is the proportion of the population requiring rehabilitation therapy. Therefore, whether the required resources can be provided in a timely manner is a major concern. In our experience, although the demand for rehabilitation services has increased, policies that limit total expenditure had forced hospitals to freeze hiring at their children’s rehabilitation centers between 2006 and 2010. Consequently, children with autism cannot easily access the services provided by the centers, and even when they can access the services, other children’s needs are prioritized. Thus, the number of children with autism on the waiting list for treatment is increasing, as are waiting times, meaning that children are losing precious treatment time.

The prevalence of autism and the accuracy of diagnosis slightly differ by country. A 2003 World Health Organization report stated that the worldwide prevalence of autism was between 1‰ and 6‰; it has been estimated to be between 2.8‰ and 4.1‰ in Taiwan [4] and 4.5‰ in Europe [5]. By 2002, in the United States, the appraised autism prevalence was 6‰–7‰ [6–8]. More recent studies have reported an increased prevalence of 11‰ in the United States [9, 10] and 11.6‰ in the United Kingdom [11].

We review the studies on the use of medical resources for autism can adopt one of two perspectives: (1) longitudinal studies on the use of medical expenditure and (2) studies on the difference in the use of resources by people with autism and those with other conditions.

Studies have examined the increase in the incidence of autism. The annual growth in the number of children with autism in Taiwan has increased substantially; the number of children with autism aged 3–12 years increased from 2071 in 2002 to 4893 in 2010 [12]. In the special education system, the number of children with autism aged 3–12 years increased from 4541 in 2007 to 5446 in 2010 [13].

In the United States, Medicaid data indicate that the population of children with autism grew by approximately 30% between 2000 and 2003. Similar growth was seen in the number of patients, prevalence rate, and overall costs; however, the average cost per person increased only slightly (approximately 3%) [14]. One study in the United States determined that the average healthcare expenditure for an individual with ASC increased by 20.4%, from US$4965 per patient in 2000 to US$5979 per patient in 2004; total expenditure for ASC increased by 142.1% over this 5-year period [3].

Another US study that used nationwide data acquired from the Medical Expenditure Panel Survey (MEPS) associated ASC with increased healthcare costs of US$3020 and increased aggregate nonhealthcare costs of US$14,061, including higher school costs of US$8610 [15].

Employing data that were sourced from the National Health Insurance (NHI) program of Taiwan, Kuan et al. [16] examined how outpatient healthcare resources were used by patients with autism during 2000–2005. During this period, autism-related visits increased from 2819 to 5273, and the number of applications for health insurance increased by 322.6%. More males applied for insurance than did females; regarding age, most applications were for children between 6 and 11 years of age. Patients with autism were more likely to use private hospitals (42.6%). Regarding the medical department visited, people visited rehabilitation departments most (46.7%), followed by the psychiatric (38.7%) and pediatric (10%) divisions. In summary, the number of children and adolescents with autism and the number of visits increased annually from 2000 to 2005, which also indicates an increase in medical utilization and resource consumption by each child.

Another NHI-based study investigated healthcare utilization from 2008 to 2011 among children without and with ASC, matched at a 3:1 ratio. On average, children with ASC had 14.2 physician visits each year; they were also more likely to be hospitalized or to visit the emergency department (ED). Consequently, children with ASC had relatively higher annual expenditure associated with physician visits, ED visits, and hospitalization as well as higher overall healthcare expenditure [17].

On the basis of 1994–1999 Medicaid data from Allegheny County of Pennsylvania, United States, Mandell et al. [18] determined that the overall expenditure for children with autism was more than those for children without and with intellectual disabilities by 10 and 3 times, respectively. Liptak et al. [19] used research reports from the US MEPS and National (Hospital) Ambulatory Medical Care Surveys and compared the medical treatment expenditure for autism with that for other conditions. The results indicated that autism incurred the highest costs, followed by depression, intellectual disabilities, and other conditions. The frequency of outpatient medical resource utilization among children with autistic spectrum conditions was 2–5 times that among children with other conditions. Shimabukuro et al. [20] used data for the period 1993–2003 from a large private insurance database in the United States and determined that patients with autism filed insurance applications 4–6 times more often than did patients without autism; they further associated a younger patient age with higher insurance application costs. Wang and Leslie [14] analyzed Medicaid data and found that between 2000 and 2003, among patients with psychiatric disorders, the average resource use by patients with autism was the third highest, behind patients with psychosis and those with intellectual disability.

Families raising children who had private insurance were more than five times as likely to incur out-of-pocket spending compared with those with children with autism who had public insurance. The commonest out-of-pocket expenditure in the United States was for medication, outpatient services, and dental care [21]. Additionally, children with ASC who had private insurance were significantly less likely than their publicly insured counterparts to receive therapy [22]. The aforementioned findings reveal gaps between public and private insurance coverage for health service use among children with ASC. Whether the NHI program in Taiwan can benefit children with ASC more requires further investigation.

The continuous growth in the population of patients with ASC poses a substantial challenge in delivering rehabilitation treatment. Insufficient manpower for the rehabilitation of children with ASC is a growing problem. The present study is the first to explore the relationship between outpatient rehabilitation care utilization and the corresponding medical costs of 3- to 12-year-old children with ASC; to this end, this study used 2008–2010 NHI data to examine the parameters associated with the resource utilization.

## Methods

NHI claims data pertaining to age; yearly outpatient care expenditure (i.e., yearly universal outpatient fees); average outpatient care expenditure; discharge diagnosis executed in accordance with the International Classification of Diseases, Ninth Revision, Clinical Modification (ICD-9-CM) coding system; medical care setting; sex; location; and finally number of outpatient rehabilitation visits (number of patients × outpatient frequency) for the period 2008–2010 were analyzed. Adults and children who persistently and consistently present specific characteristic behaviors—for example, repetitive and stereotypical patterns of restricted behaviors, interests, and activities as well as qualitative impairment in social communication and interaction—are diagnosed as having ASC. In Taiwan, children with mental developmental problems are on average first diagnosed at 2.7 years of age. However, definite diagnosis for autism and early intervention for treatment are received on average 3.7 years later [4]. Adolescents with autism who are older than 12 years, compared with younger children with autism, have lower outpatient medical expenditure and lower utilization of rehabilitation resources but higher medicine costs [20]. Therefore, the present research work’s scope was limited to children in the age range 3–12 years having a minimum of one outpatient rehabilitation claim, that is, ICD-9-CM autism diagnostic codes 299–299.91 recorded for billing after diagnosis by qualified doctors. Per the presence or absence of these codes, the data of the children who used rehabilitation resources were divided into two groups: autism with catastrophic illness card and nonautism (hereafter, the ASC and non-ASC groups, respectively). Children with catastrophic illness card has the accurate diagnostic validity. A series of professional review of application is required. ASD instead of ADHD can apply for the catastrophic illness card. So we choose the children with catastrophic illness card of ASD to decrease the opportunity of comorbidity. We compared the rehabilitation resource utilization in the ASC and non-ASC groups to determine the influencing factors.

SPSS Statistics 17.0 version(SPSS Inc., Chicago, IL, USA) was executed to analyze the entirety of the data in this study. Patient demographics, rehabilitation care expenditure, and medical care features in the ASC as well as non-ASC groups were compared through χ^2^ testing, whereas outpatient care expenditure and outpatient rehabilitation frequency in the ASC group were evaluated through *t* testing. In addition, the factors associated with rehabilitation care expenditure and visit frequency in the ASC group were determined through multiple linear regression model analyzing natural logarithm of expenditures and negative binomial model for visit frequency. We controlled individual factors and medical facility factors where patients seeking care into regression analysis. Confidence intervals (CIs) around all mean values and regression coefficients were estimated using nonparametric procedures.

We established the following three hypotheses: (1) The availability of rehabilitation services for children with autism cannot keep pace with growth in the number of children with autism. (2) The numbers of rehabilitation services available for patients with and without autism differ significantly. (3) The costs associated with rehabilitation differ significantly for patients with and without autism.

Before release for research, National Health Insurance Research Database (NHIRD) data are anonymized. Thus, the current research work was in compliance with the standards of the 2004 Declaration of Helsinki, in addition to being ratified by the Institutional Review Board of Antai Tian-Sheng Memorial Hospital (17–087-B) in Taiwan.

## Results

Table 1 shows the 2008–2010 NHI data of the study population, namely children who were determined to be 3–12 years in age and to use outpatient rehabilitation care. The ASC group exhibited an annual growth in the proportion of children availing rehabilitation treatments, with 20.1 (mean) annual rehabilitation care visits; each of these visits cost on average 2782.3 points (1 point equals approximately NT$1), and the average annual cost per person was approximately 54,725.2 points. In all years, compared with the non-ASC group, medical expenditure in the ASC group was almost 3 times higher (*p* < 0.001), and rehabilitation utilization was more widely distributed in the ASC group (*p* < 0.001).

**Table 1:** Rehabilitation resource utilization among 3–12-year-old children (NHIRD data)

Year	ASC (*n* = 3227)			Non-ASC (*n* = 150,451)		
	2008	2009	2010	2008	2009	2010
Number (%)	1085 (32.3)	1053 (32.0)	1085 (2.0)	47,758 (97.8)	50,553 (98.0)	52,140 (98.0)
Average of frequency (SD)^b^*	18.76 (15.45)	20.87 (16.12)	20.52 (15.59)	6.96 (9.54)	7.13 (9.66)	7.08 (9.42)
Average cost/claim point^a^ (SD)^b^*	2761.63 (1717.63)	2803.29 (1730.81)	2781.94(1701.41)	2521.48 (1856.24)	2559.97 (1855.96)	2578.44 (1847.73)
Average total annual cost/claim point^a^ (SD)^b^*	55,953.06 (52,064.89)	51,698.48 (51,698.48)	50,706.14 (50,131.40)	17,521.16 (33,278.63)	18,251.02 (33,636.77)	18,245.37 (32,676.85)

Average frequency: total annual outpatient rehabilitation visits/annual number of patients

Average cost/claim point: average universal outpatient claim

Average total annual cost/claim point: Average cost × average frequency per patient

*p < 0.001 (comparing the overall mean for the 3 years in the ASC and non-ASC groups)

^a^1 claim point equals approximately NT$1

^b^*SD* standard deviation

Table 2 lists the demographics of the study population, which was predominantly male (*p* < 0.001), with an approximate boy:girl ratio of 5:1. The average rehabilitation resource utilization was relatively higher among school-aged children; however, over time, the proportion of preschool children increased to almost equal that of school-age children (*p* < 0.001).

**Table 2:** Demographics of 3–12-year-old children receiving rehabilitation care

Year	ASC			Non-ASC		
	2008	2009	2010	2008	2009	2010
	*n* = 1089	*n* = 1053	*n* = 1053	*n* = 47,758	*n* = 50,553	*n* = 52,140
Age (%)*						
Preschool	37.7	37.5	37.5	47.5	49.0	50.6
School	62.3	62.5	62.5	52.5	51.0	49.4
Gender(%)*						
Boys	83.1	83.2	83.7	64.8	64.8	64.6
Girls	16.9	16.8	16.3	35.2	35.2	35.4
Male/Female Ratio	4.9	5.0	5.1	1.8	1.8	1.8

*p < 0.001 (comparing the overall mean for the 3 years in the ASC and non-ASC groups)

As shown in Table 3, which characterizes the rehabilitation setting, patients with ASC were less likely to use rehabilitation resources at medical centers (10.43%; *p* < 0.001) and were more likely to visit nonteaching hospitals than teaching hospitals (59%; *p* < 0.001). Most (48%) rehabilitation resource providers are located in Taipei (*p* < 0.001).

**Table 3:** Characteristics of rehabilitation resource settings

Year	ASC			Non-ASC		
	2008	2009	2010	2008	2009	2010
Medical setting(%)*						
Medical center	8.0	11.6	11.7	16.6	17.0	16.3
Metropolitan hospital	32.2	26.7	27.3	35.0	33.8	35.1
Local Hospital	28.5	31.0	31.6	21.8	23.4	24.1
Clinic	31.3	30.7	29.4	26.7	25.8	24.5
Teaching hospital(%)*						
Yes	42.6	40.2	40.3	55.1	53.9	54.6
No	57.4	59.8	59.7	44.9	46.1	45.4
Location(%)*						
Taipei	40.7	46.5	51.1	29.8	32.8	43.8
North	23.7	20.7	19.4	19.8	19.2	19.4
Central	11.5	9.7	9.3	22.5	21.7	14.1
South	8.2	8.1	7.0	13.9	12.9	7.9
Kaohsiung and Pingtung	15.6	14.5	12.8	12.1	11.7	13.2
East	0.3	0.4	0.5	1.8	1.7	1.5

Location: Taipei is the largest city in Taiwan and the city with the most healthcare resources. The East region is the most rural area and the region with the fewest healthcare resources

*p < 0.001 (comparing the overall mean for the 3 years in the ASC and non-ASC groups)

As shown in Table 4, which summarizes the negative binomial model and multiple linear model results, elements outlined to be associated with expenditure related to rehabilitation—for example, demographics as well as properties of relevant hospital, year of resource utilization—significantly influenced medical expenditure and frequency of visits (*p* < 0.01).

**Table 4:** Regression models of rehabilitation use and expenditure

Variable(reference)	Average of frequency^a^ IRR (95%CI)	ln(Average annual cost)^b^ β (95%CI)	ln(Average total annual cost)^b^ β (95%CI)
Constant	14.054 (9.615–20.542)**	10.024 (9.650–10.398)**	7.492 (7.328–7.656)**
Year(2008)			
2009	1.123 (1.053–1.197)**	0.192 (0.097–0.287)**	0.033 (− 0.001–0.066)
2010	1.116 (1.047–1.190)**	0.133 (0.035–0.230)*	0.013 (− 0.021–0.046)
Age(preschool age)			
School age	0.812 (0.768–0.858)**	− 0.350 (− 0.433- -0.268)* *	− 0.144 (− 0.172- -0.116)**
Gender (girl)			
Boy	1.009 (0.941–1.083)	− 0.074 (− 0.180–0.031)	− 0.078 (− 0.115- -0.041)**
Medical setting(Medical Center)			
Metropolitan hospital	1.231 (1.128–1.344)**	0.334 (0.205–0.462)**	0.128 (0.079–0.177)**
Local hospital	1.139 (0.996–1.304)	0.457 (0.264–0.650)**	0.337 (0.268–0.406)**
Clinic	1.188 (1.026–1.376)*	0.196 (− 0.015–0.406)	0.049 (− 0.026–0.123)
Teaching hospital(no)			
Yes	0.717 (0.638–0.805)**	−0.278 (− 0.443- -0.113)**	0.080 (0.022–0.138)**
Location(East)			
Taipei	1.397 (0.983–1.987)	0.384 (0.067–0.700)*	0.269 (0.118–0.420)**
North	1.687 (1.182–2.409)**	0.579 (0.251–0.907)**	0.284 (0.130–0.437)**
Central	1.512 (1.056–2.165)*	0.247 (−0.088–0.582)	0.064 (− 0.092–0.221)
South	1.434 (0.999–2.059)	0.228 (−0.112–0.568)	0.067 (− 0.094–0.228)
Kaohsiung and Pingtung	1.704 (1.190–2.441)**	0.625 (0.290–0.959)**	0.307 (0.152–0.461)**

Average frequency: total annual outpatient rehabilitation visits/annual number of patients

Average cost/claim point: average universal outpatient claim

Average total annual cost/claim point: Average cost × average frequency

^a^Negative binomial model reporting incidence-rate ratios

^b^Natural logarithm of costs using multiple linear regression

*p < 0.05

**p < 0.01

## Discussion

The use of healthcare services by patients with autism is projected to continue to grow because of the increasing prevalence of autism. Compared with the non-ASC group, the noted increase in single payouts was more significant in the ASC group, with the corresponding *p* value being < 0.001. However, average single payment increased by only 21.35 points (1 claim point equals approximately NT$1), an increase of 7.5‰between 2008 and 2009. Although the points continued to increase between 2009 and 2010, the increase was nonsignificant.

Similarly, the average frequency of outpatient visits of patients with ASC increased between 2008 and 2009 but slightly decreased between 2009 and 2010. This finding is inconsistent with that of Kuan et al. [16], who reported a significant increase in health insurance payment points between 2000 and 2005 in Taiwan. Moreover, in America, Mandell et al. [18] determined that the average resource expenditure of patients with autism changed by a similar amount between 1994 and 1999 annually and that this increase was influenced by the policies and finances of Allegheny County, Pennsylvania. Furthermore, scholars such as Wang and Leslie [14] have studied the expenditure of children with autism who used Medicaid in the United States and have determined that the average cost per person increased only modestly (3%) between 2000 and 2003. This mentioned finding is consistent with the results derived in our research work. This may be because of the stable growth in the use of rehabilitation resources in Taiwan, which in turn is attributable to the continual growth in the population of patients with autism since 2000. However, in recent years, rehabilitation resources have become saturated, which along with the controls on the total hospital expenditure may have stopped the growth in the resources available for autism support groups; this may have limited resource utilization among people with autism.

This study also investigated the use of healthcare and rehabilitation resources among patients without and with autism; patients with autism spent approximately 3 times the amount that people without autism spent. Wu et al. [17] revealed that the mean annual physician visit expenditure and total health expenditure of children with autism were 3 and 3.8 times those of children without autism, respectively in Taiwan. In America, Mandell et al. [18] determined that the overall medical expenditure of patients with autism is 3–10 times that of patients without autism. Liptak et al. [19] determined that the medical expenditure of patients with autism is 3–6 times that of patients with other diagnosis, which agrees with the findings of this study. Shimabukuro et al. and Tara A. [14, 20] compared autism and nonautism groups and presented results that are consistent with those of our study. Patients with autism spend 4–6 times as much on private health insurance as patients without autism, which supports our hypothesis that the invested medical resources of patients with and without autism differ significantly.

This study revealed that each patient with autism visits hospital outpatient departments 20 times per year; this result is similar to that of another study that revealed that children with ASC had 14.2 physician visits annually [17]. By contrast, Kuan et al. [16] revealed that from 2000 to 2005, on average, each patient with autism visited hospital outpatient departments 7–12 times per year. They also claimed that the number of applications would continue to grow; this explains the higher number we found for 2008–2010 in Taiwan. Studies in other countries vary in their findings. Liptak et al. [19] stated that patients with autism utilized medical services approximately 41.5 times per year per person, which is considerably higher than that in this study. In America, children with parent-reported ASD had higher levels of health care office visits compared with children without ASD [14]. This may be because in our study, the number of visits refers to the number of outpatient visits for rehabilitations and a single NHI entry may include several rehabilitation treatment items (e.g., occupational, physical, and speech therapy as well as psychotherapy). Thus, in this study, the number of visits does not correspond to the actual services received. Access to this detailed information via prescription and medical treatment records might make it possible to obtain more accurate results.

The ratio of boys to girls with autism in this study was approximately 5:1, which agrees with those in other studies [2, 10, 17, 23]. This study revealed no significant differences between boys and girls in terms of visit frequency and annual medical expenditure, indicating that sex is not a predictor for utilization of medical resources. This is in agreement with Kuan et al. [16]. This study determined that sex does not predict medical costs and that parents of children with autism are unaffected by sex differences in terms of their children’s treatment intensity.

Preschool patients with autism were significantly more likely to use rehabilitation resources than were school-age children. This result is similar to that of Shimabukuro et al. [20] and that of Wu et al. [17]. The latter found that insurance costs before 5 years of age were higher than those after 5 years; in particular, outpatient expenditure noted for patients younger than 5 years was higher than that noted for patients in other age groups. No studies have identified the factors that affect rehabilitation resource use among preschool children, but this may be because governments appropriate more welfare spending for preschool children (e.g., rehabilitation transportation grants). This makes parents of preschool children to be more willing to use rehabilitation resources.

Finally, this study determined that patients with autism with catastrophic illness cards used rehabilitation resources more than did all other types of the studied patients. This is in agreement with Kuan et al. [16]. Having a catastrophic illness card has a significant effect on the cost of care; this card exempts patients from payments for relevant medical treatments (e.g., rehabilitation therapy). Patients with a diagnosis of ASC can apply for a disability card. Patients with disability cards must pay partial medical costs. Having a catastrophic illness card incentivizes the active use of rehabilitation services.

Regarding hospital characteristics, hospital location was associated with single health insurance application; most payments were made to district hospitals, followed by regional hospitals, primary care clinics, and medical centers. Visit frequency was higher at primary care clinics and lower at district and regional hospitals as well as medical centers. Patient expenditure was highest at district hospitals, followed by primary care clinics, regional hospitals, and medical centers. This indicates that district hospitals and primary care clinics have a pivotal role in the utilization of rehabilitation resources by patients with autism. Notably, patients attending medical centers and regional hospitals pay the most to the NHI bureau, and these payments are significantly higher than those to district hospitals and primary care clinics. This finding indicates that local hospitals and primary clinics provide more intensive services, offer more relevant rehabilitation programs, and are more accessible than are regional hospitals and medical centers. These results agree with Kuan et al. [16].

Patients who have catastrophic illness cards and comorbid conditions were more likely to visit district hospitals and primary care clinics to access rehabilitation resources. Only patients with disability cards were more likely to visit regional hospitals and medical centers. Taipei City, constituting Taiwan’s biggest city and being one with the most healthcare resources, saw the highest resource supplementation, followed by Northern Taiwan, Central Taiwan, the Kaohsiung–Pingtung area, Southern Taiwan, and Eastern Taiwan. Regarding annual and average costs as well as average visits, Northern Taiwan, Southern Taiwan, and the Kaohsiung–Pingtung area saw a significant increase in medical resource utilization relative to Eastern Taiwan (most of which comprises rural areas with relatively few healthcare resources). Despite having the highest observed rehabilitation resource utilization, Taipei City’s average total cost and average number of visits did not differ significantly from those in Eastern Taiwan. This may be because children with ASC in Taipei City may have had early detection and may have received treatment for relatively milder symptoms. Furthermore, parents in Taipei City can opt for a wider selection of non–NHI-related therapies or treatments, with out-of-pocket payments. Thus, since they have more treatment alternatives, children in Taipei have more favorable outcomes, requiring less frequent follow-up rehabilitation visits.

This study has certain drawbacks. First, this study discussed the differences in the use of resources for autism. However, our data were from a 3-year period and lack long-term follow-up. Second, this study used data from the NHI database, which is constrained by the unavailability of inpatient treatment data as only rehabilitation outpatient treatment data are available in the database. Moreover, the rehabilitation data did not include diagnoses made by other specialists, such as pediatricians, psychiatrists, and child psychiatrists. Because of health insurance applications, healthcare providers differ in payment standards. Third, if a database entry on outpatient rehabilitation service contains six distinct treatment dates, it does not represent the treatment intensity and frequency. The proportion of professionals and payments differ. For example, many treatments, especially speech therapy, provided for children with autism with delayed speech progress cannot individually be analyzed for resource utilization. Fourth, children with ASC often have comorbid conditions: 47% of children with ASC had at least one comorbid condition [14]. We could not clarify the severity and comorbidity of ASC. Fifth, this research work introduces a secondary analysis of data derived from a National Institutes of Health–maintained database. This database inherently has a deficiency of data on patients’ as well as their parents’ education background, readiness to pursue treatment, quality of family interaction, social status, expenditure restrictions, and family income. Moreover, the database contains information on the insurance payments but not the actual income of the families. Nevertheless, this study employed nationally representative datasets for this analysis and can provide insights into resource utilization among children with ASC, thus making valuable contributions to the literature.

## Conclusions

In Taiwan, few studies have examined the utilization of nonpharmacological treatments as rehabilitation resources. The condition of patients with autism varies greatly. Rehabilitation necessitates professional personnel and is time-intensive. District hospitals and primary care clinics serve more patients with autism, but their expenditure on autism resources is less than 50% that of regional hospitals and medical centers. Because of these restrictions, the availability of services for patients with autism has decreased. Government policies may be necessary to narrow the expenditure gap between hospital types and to induce incentives for operators to devote more resources to meet the needs of children with autism. Another concern in Taiwan is the uneven distribution of medical resources, as access to resources in remote areas remains low: Taipei City has the most resources, whereas the East has the fewest. This disparity decreases the willingness to supplement healthcare services. In summary, such inadequate access to rehabilitation strongly limits the available treatment avenues for children with ASC to address educational and medical problems. Thus, for reducing future burdens placed on families, society, and the education system, government agencies must implement effective policies by adequately assisting the affected children in addition to their families.

## Data Availability

Data are available from the NHIRD. Because of legal restrictions imposed by the government of Taiwan in relation to the Personal Information Protection Act, data cannot be made publicly available. Requests for data can be sent as a formal proposal to the NHIRD (http://nhird.nhri.org.tw).

## Abbreviations

ASC: Autism spectrum condition
